# Paraneoplastic Neurological Syndromes as Initial Presentation of Tumors: An Eight-Year Single-Center Experience

**DOI:** 10.3390/jcm13030824

**Published:** 2024-01-31

**Authors:** Konstantinos Melanis, Maria-Ioanna Stefanou, Dimitrios K. Kitsos, Athanasia Athanasaki, Aikaterini Theodorou, Eleftheria Koropouli, Anna Keramida, Evangelia Makrina Dimitriadou, Dimitrios Tzanetakos, Elizabeth Andreadou, Ioanna Koutroulou, Sotirios Giannopoulos, George P. Paraskevas, Georgios Tsivgoulis, John S. Tzartos

**Affiliations:** 1 Second Department of Neurology, “Attikon” University Hospital, School of Medicine, National and Kapodistrian University of Athens, 12462 Athens, Greece; melaniskos@gmail.com (K.M.); dkitsos@icloud.com (D.K.K.); athanasia.athan@yahoo.gr (A.A.); katetheo24@gmail.com (A.T.); ekoropou@med.uoa.gr (E.K.); akeramida@outlook.com (A.K.); evadim93@hotmail.gr (E.M.D.); dtzanetakos@med.uoa.gr (D.T.); sgiannop@uoi.gr (S.G.); gtsivou@med.uoa.gr (G.T.); jtzartos@med.uoa.gr (J.S.T.); 2 First Department of Neurology, “Eginition” University Hospital, School of Medicine, National & Kapodistiran University of Athens, 12462 Athens, Greece; eandread@med.uoa.gr; 3 Second Department of Neurology, AHEPA University Hospital, School of Medicine, Faculty of Health Sciences, Aristotle University of Thessaloniki, 54636 Thessaloniki, Greece; ikoutroulou@yahoo.com

**Keywords:** paraneoplastic neurological disorders, paraneoplastic antibodies, limbic encephalitis, paraneoplastic diagnostic criteria, PNS-CARE

## Abstract

Background: Paraneoplastic Neurological Syndromes (PNS) comprise a diverse group of disorders propagated by immune-mediated effects of malignant tumors on neural tissue. Methods: A single-center longitudinal study was performed including consecutive adult patients treated at a tertiary academic hospital between 2015 and 2023 and diagnosed with PNS. PNS were ascertained using the 2004 and the revised 2021 PNS-Care diagnostic criteria. Results: Thirteen patients who fulfilled the 2004 definite PNS criteria were included. PNS comprise diverse neurological syndromes, with neuromuscular junction disorders (54%) and limbic encephalitis (31%) being predominant. PNS-related antibodies were detected in 85% of cases, including anti-AChR (*n* = 4), anti-P/Q-VGCC (*n* = 3), anti-Hu (*n* = 3), anti-Yo (*n* = 1), anti-Ma (*n* = 1), anti-titin (*n* = 1), anti-IgLON5 (*n* = 1), and anti-GAD65 (*n* = 1). Thymoma (31%), small-cell lung cancer (23%), and papillary thyroid carcinoma (18%) were the most frequent tumors. Imaging abnormalities were evident in 33% of cases. Early immunotherapy within 4-weeks from symptom onset was associated with favorable outcomes. At a mean follow-up of 2 ± 1 years, two patients with anti-Hu and anti-Yo antibodies died (18%). Four and three patients fulfilled the 2021 PNS-Care diagnostic criteria for definite and probable PNS, respectively. Conclusions: This study highlights the clinical heterogeneity of PNS, emphasizing the need for early suspicion and prompt treatment initiation for optimal outcomes.

## 1. Introduction

Paraneoplastic neurological syndromes (PNS) constitute a diverse group of disorders that result from remote effects of malignant neoplasms, distinct from direct invasion [1,2]. Current diagnostic algorithms approach PNS as disorders that (i) are frequently linked to distinct clinical phenotypes, potentially affecting any part of the human nervous system; (ii) are typically present in the setting of cancer; and (iii) are of presumed immunological origin [3]. With respect to the latter, PNS are frequently associated with circulating antibodies (PNS-Abs), that propagate immune-mediated responses [4]. Notably, these antibodies have been previously characterized as “onconeural” to designate shared antigenic targets between neuronal tissue and tumors. Yet, the fact that concurrent malignancy is not always present, while homology between neuronal and tumor antigenic epitopes cannot always be established, has led to the gradual abolition of this term in current literature.

Since their first description, the spectrum of PNS-Abs continues to expand, with the shift in diagnostic approaches reflected in the recent update of PNS diagnostic criteria with the incorporation of updated recommendations for PNS-Abs laboratory testing [5]. In clinical practice, identification of PNS-Abs facilitates PNS diagnosis. Nevertheless, due to the variable sensitivity and specificity of PNS-Abs testing, their presence is not obligatory for establishing PNS. In addition to laboratory testing, and in accordance with current diagnostic criteria (Table 1), two further axes should be explored when suspecting PNS: (i) evaluation of clinical phenotype and (ii) rigorous tumor assessment in order to establish a definite, probable, or possible PNS accordingly. Clinically, these syndromes are typically characterized by subacute symptom onset, progressing disability, and limited response to immunotherapies. Although diverse clinical features and a broad differential diagnosis are associated with PNS, distinct clinical phenotypes may prompt specific antibody testing and targeted tumor search as per current guidelines.

From a pathophysiological perspective, it is also noteworthy, that the heterogeneity of PNS has been linked to the wide distribution of antigenic epitopes across the nervous system, involving both the central and peripheral neuro-axis [6]. With respect to prognosis, early suspicion of PNS is decisive, as PNS may be the sole clinical manifestation of an underlying tumor, often preceding tumor diagnosis by several years [7]. In addition, recent evidence indicates that the identification of specific PNS-Abs may significantly correlate with a clinical prognosis [8].

The aim of the present study was to comprehensively characterize clinical phenotypes, paraclinical findings, treatment responses, and clinical outcomes for PNS, analyzing consecutive patients treated within an 8-year period at an academic tertiary referral hospital. In addition, we sought to examine the comparative diagnostic efficacy of the 2004 and the revised 2021 PNS-Care diagnostic criteria in this patient population [3,5].

## 2. Methods

### 2.1. Ethical Approval and Patient Consent

This study was approved by the Ethics Committee of our Institution (Protocol number: EBD185/5-4-2022). All patients provided written informed consent for study participation and use of de-identified data was granted in accordance with ethical requirements as stated in the Declaration of Helsinki in its currently applicable form [9].

### 2.2. Autoantibody Detection

Patient sample testing for paraneoplastic disorders, including cerebrospinal fluid (CSF) and serum, was conducted at Tzartos NeuroDiagnostics laboratory (ISO 9001-2015) using standardized methods. Immunohistochemistry, cell based assay (CBA) and dot-blot analysis for antigens related to autoimmune and paraneoplastic disorders were performed [10,11]. With Immunohistochemistry, serum at dilution 1/10 (or CSF, undiluted) was incubated with monkey brain cerebellum frozen tissue, followed by incubation of the treated tissue with a secondary FITC-labelled anti-human IgA GM antibody (Euroimmun, Lubeck, Germany; FA1111-1005), as described by the manufacturer, for visualization. For CBA, serum at dilution 1/10 (or CSF, undiluted) was incubated with fixed HEK cells transfected with various antigens (e.g., NMDA, Caspr2) followed by incubation with a secondary FITC-labelled anti-human IgG antibody (Euroimmun, Lubeck, Germany; FA1111-1005), as described by the manufacturer, for visualization. For dot-blot, patient serum was incubated at 1/100 dilution (or CSF, undiluted), against a panel of 12 antigens (Tr/DNER, GAD65, Zic4, Titin, SOX1, Recoverin, Hu, Yo, Ri, PNMA2/Ta, CV2, Amphiphysin) followed by incubation with a secondary anti-IgG conjugated with alkaline phosphatase, followed by the addition of a substrate for visualization, as described by the manufacturer (Euroimmun, Lubeck, Germany; DL 1111-1601-7G). In all patients, PNS-Abs were consistently identified utilizing both immunohistochemistry and dot-blot analysis techniques.

### 2.3. Setting and Diagnostic Criteria

The present study was conducted at a tertiary academic referral hospital in Athens (Second Department of Neurology of the National & Kapodistrian University of Athens, “ATTIKON” University Hospital) including data collated over an eight-year study period (2015–2023). We retrospectively reviewed the medical records of patients treated at our center during the study period and included those who fulfilled the criteria of definite PNS based on the 2004 diagnostic criteria [3].

The patients included in the cohort fulfilled the criteria established in 2004 for PNS, presenting with: (1) classical syndromes characterized by the development of cancer, within 5 years of their initial diagnosis; (2) non-classical syndromes diagnosed within 5 years of a cancer diagnosis, with the presence of PNS-Abs; (3) non-classical syndromes showing substantial neurological improvement following cancer treatment; or (4) either classical or non-classical syndromes in conjunction with the identification of well-characterized PNS-Abs (anti-Hu, Yo, CV2, Ri, Ma2, or amphiphysin) [3]. In accordance with previous literature, “classical syndromes” encompassed neurological syndromes frequently associated with cancer, including encephalomyelitis, limbic encephalitis (LE), rapidly progressive cerebellar syndrome, opsoclonus-myoclonus, sensory neuronopathy, gastrointestinal pseudo-obstruction (enteric neuropathy), and Lambert–Eaton myasthenic syndrome (LEMS) [12,13]. By contrast, “non-classical syndromes” encompassed poorly characterized PNS manifestations [12,13]. In addition, patients with myasthenia gravis (MG) with underlying thymoma were also included, based on the European Federation Neurological Society guidelines for PNS [14,15,16].

The 2021 PNS-Care criteria were either prospectively or retrospectively applied to evaluate their comparability with the 2004 PNS criteria (Table 1). These criteria utilize a scoring system as a recently developed tool to assess and diagnose paraneoplastic diseases [5]. The 2021 PNS-Care score integrates several elements, encompassing the risk associated with specific clinical phenotypes (i.e., previously characterized as “classical” or “non-classical syndromes” based on the 2004 criteria). These phenotypes are now stratified as either high risk or intermediate risk based on the recognition of highly pathognomonic features, such as encephalomyelitis, limbic encephalitis, and sensory neuronopathy, among others. Additionally, the scoring system considers the identification of PNS-Abs, categorizing them as high (>70% association with cancer), medium (30–70% association with cancer), and low (<30%) risk. Furthermore, the presence of cancer consistent with the identified antibody within 2 years of presentation is taken into account [5].

Paraclinical findings including chest, abdominal computed tomography, testicular ultrasound, upper gastrointestinal endoscopy, colonoscopy, magnetic resonance imaging (MRI) of the brain and spine, nerve conduction studies, electromyography and positron emission tomography (PET), along with clinical and long-term follow-up data were collated and reviewed for the present analysis. Malignancy was pathologically ascertained in all cases.

### 2.4. Statistical Analysis

For continuous data, the means ± standard deviation (SD) are reported. Categorical variables are summarized by counts and percentages. The Shapiro–Wilk test was utilized to assess the normality of variables. For normally and non-normally distributed variables, the mean ± standard deviation (SD) or median and the corresponding interquartile range (IQR) were estimated, respectively.

## 3. Results

### 3.1. Clinical Characteristics and PNS Phenotypes

A total of 13 patients who fulfilled the 2004 diagnostic criteria for definite PNS were included (Table 2). The mean age at onset was 63 years (standard deviation [SD]: 14, with a male to female ratio of 7:6. In all cases, alternative diagnoses (e.g., autoimmune encephalitis, autoimmune myopathies, viral encephalitis, and rapidly progressive dementias) were excluded prior to PNS diagnosis. The spectrum of PNS manifestations included: LE (limbic encephalitis) (31%, 4/13), MG (31%, 4/13), Lambert–Eaton myasthenic syndrome (LEMS) (23%, 3/13), cerebellar ataxia (CA) (15%, 2/13), and peripheral neuropathy (PN) (8%, 1/13). In patient #6 (Table 2), there was an overlap of two neurological syndromes (LE with LEMS). Among the observed symptoms of LE were epilepsy (50%, 2/4), cognitive dysfunction (50%, 2/4), and parasomnia (25%, 1/4). Regarding the four patients with LE, patient #2 exhibited anti-Ma2 antibodies, patient #3 displayed anti-ΙgLON5 antibodies, patient #10 showed GAD65 antibodies, while patient #6, who presented clinical overlap with LEMS, expressed both anti-Hu and anti-P/Q-VGCC (voltage-gated calcium channel) antibodies (Table 2). Among patients diagnosed with MG, all demonstrated anti-AchR (acetylcholine receptors) antibodies, with patient #1 showing co-expression of anti-titin (Ti) antibodies (Table 2). Concerning the two patients with LEMS, patient #4 expressed anti-P/Q-VGCC while patient #6 exhibited antibody overlap with anti-Hu and anti-P/Q-VGCC.

According to the aforementioned phenotypes, three individuals demonstrated a rapid symptom progression. Notably, patient #6, with the overlap syndrome of LEMS and LE with anti-Hu and anti-P/Q-VGCC antibodies, exhibited a rapid progression within a week. Patient #9, presenting with peripheral neuropathy and anti-Hu antibodies, experienced a rapid progression over a span of two weeks. Patient #10, presenting with LE with anti-GAD65 antibodies, demonstrated a rapid clinical presentation over a period of three weeks (Table 3).

### 3.2. PNS-Related Antibodies

**Figure 1:**
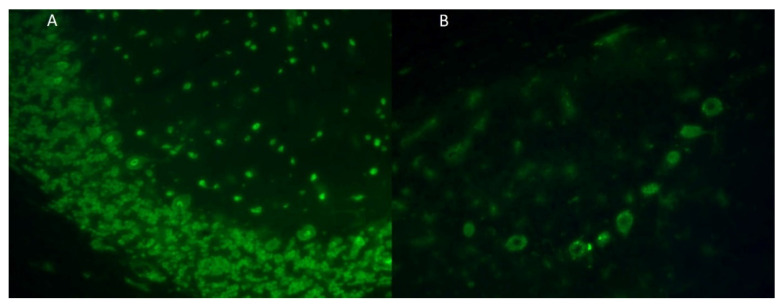
Immunohistochemistry images demonstrating serum binding of patient #6 and patient #9 to monkey brain cerebellum. Immunostaining following incubation of patient’s #6 serum and patient’s #9 serum on monkey brain cerebellum shows: granular and Purkinje neurons (**A**), with dot-blot analysis exhibiting anti-Hu antibodies, and Purkinje neurons (**B**), with dot-blot analysis exhibiting anti-Yo antibodies.

PNS-Abs were identified in eleven patients (85%): anti-AChR (acetylcholine receptor) (*n* = 4, 31%), anti-P/Q-VGCC (*n* = 3, 23%), anti-Hu (*n* = 3, 23%), anti-Yo (*n* = 1, 8%), anti-Ma2 (*n* = 1, 8%), and anti-striational (particularly anti-Ti) (*n* = 1, 8%) (Figure 1). The other two patients expressed anti-IgLON5 and anti-GAD65 antibodies that are considered mostly autoimmune and were included due to fulfillment of the 2004 PNS criteria. In two cases, the overlap of two PNS-Abs was recognized (one patient with both anti-Hu and anti-P/Q-VGCC, and another patient with anti-striational (anti-Ti) and anti-AChR (Table 2)). All antibodies were detected in CSF and serum except those found only in serum in patients with MG and LEMS.

### 3.3. Tumors Associated with PNS

The underlying tumors are shown in Table 2. The most frequently detected tumor was thymoma (31%, 4/13), followed by small-cell lung cancer (23%, 3/13). The rest were papillary thyroid carcinoma (15%, 2/13), gastric adenocarcinoma (8%, 1/13), adenocarcinoma of prostate (8%, 1/13), lymphohyperplastic cancer (8%, 1/13), and non-small-cell lung cancer (8%, 1/13). Patient #6 manifested with multiple PNS (LE and LEMS) with a single underlying tumor which was small-cell lung cancer. Regarding thymoma, the correlated antibody was AchR in all cases (100%, 4/4). In the context of small-cell lung cancer, P/Q-VGCC antibodies (67%, 2/3) and anti-Hu antibodies (67%, 2/3) were detected. Papillary thyroid carcinoma was associated with antibodies directed against Yo and Ma2, while gastric adenocarcinoma was associated with Yo antibodies. For adenocarcinoma of the prostate and for lymphohyperplastic cancer IgLON5 and GAD65 antibodies were detected, respectively. In the case of non-small-cell lung cancer, the combination of Hu and P/Q-VGCC antibodies were recorded. Underlying tumors were detected concurrently in 7 patients (54%), before the PNS in 1 (8%), and in 5 patients (38%) after the diagnosis of PNS within a six-month period. The simultaneous diagnosis of tumors and PNS were most common in neuromuscular PNS.

### 3.4. CSF Findings

CSF analysis was performed in cases presenting with LE, CA, and peripheral neuropathy (*n* = 7) (Table 2). Four patients (57%) exhibited an inflammatory CSF profile characterized by lymphocytic pleocytosis and/or a slightly elevated protein level. Additionally, the IgG index was normal, except for one patient with polyneuropathy, in which the IgG index was measured at 0.9 (with a normal cut-off of 0.8). Oligoclonal bands (OCBs) were negative in all investigated patients (*n* = 7).

### 3.5. MRI Imaging

Brain and spinal MRIs were available in all cases presenting with central nervous system involvement (*n* = 6) (Table 2). MRI abnormalities were evident in 33% (2/6 of patients). Among cases with LE, patient #6 exhibited a high signal intensity on the Fluid Attenuated Inversion Recovery (FLAIR) sequences within the left limbic area. For the diffusion weighted imaging (DWI), restricted diffusion was noted in the left medial temporal lobe. No contrast enhancement was detected on T1-weighted gadolinium-enhanced imaging. This particular case corresponds to the patient with the overlapping syndromes of LE and comorbid LEMS (patient #6 in Table 2). Subsequent MRI evaluations of this patient at three and nine months indicated a gradual improvement in imaging findings. In patient #7 with CA, a cerebral MRI revealed cerebellar atrophy. A follow-up brain MRI after six months revealed neither an improvement nor progression of the atrophy.

### 3.6. Applied Treatments

Nearly all cases (11/13, 85%) were started on immunomodulatory therapy within 4 weeks from symptom onset from neurological syndrome onset. The two exceptions were: patient #3 with LE, who was treated at 12 months after presentation due to a delayed referral (i.e., initial misdiagnosis as psychosis) and patient #2, who was diagnosed with papillary thyroid carcinoma and following tumor resection and was stabilized with antiepileptic therapy (lacosamide) not requiring immunosuppression (Table 3). Concerning immunomodulatory therapies, patients were initially treated with intravenous immunoglobulin (IVIG, 2 g/kg divided over 3–5 days: (50%, 6/12), corticosteroids (75%, 9/12), or plasma exchange (PLEX: 8%, 1/12)). Eight cases (75%) received monotherapy (3 IVIG, 6 corticosteroids) (Table 3). Three cases (25%) received combined therapy (IVIG and corticosteroids). One case received all three types of treatment (corticosteroids, IVIG, and PLEX) (Table 3). Corticosteroid treatment typically involved an intravenous administration of methylprednisolone at a daily dosage of 1 g for 3–5 days (17%, 2/12) followed by a tapering course of oral prednisone (75%, 9/12) (Table 3). Additional treatments were considered when first-line therapy failed to achieve sufficient neurological stabilization or for maintenance (Table 3). Patient #11 with MG, who was non-responsive to treatment, received cyclophosphamide as a rescue therapy and mycophenolate mofetil as a maintenance therapy (Table 3). In all MG cases, pyridostigmine was utilized as a symptomatic therapy while for the three patients with LEMS only two received treatment (1 pyridostigmine, 1 amifampridine) (Table 3). All cases underwent additional oncological treatment (12/13 tumor resection, 3 chemotherapy, and 2 radiotherapy) (Table 3).

### 3.7. Follow-Up and Clinical Outcomes

The mean elapsed duration between neurological manifestation and the diagnosis of tumors was 21 ± 8 months. Within a six-month period, tumors were diagnosed in five patients (38%) after the manifestation of PNS. Specifically, in three cases with Ma2 (1/3) and Yo antibodies (2/3), papillary thyroid carcinoma and gastric adenocarcinoma were detected. In patient #9 (Table 3), small-cell lung carcinoma was identified at three months, while in patient #10, lymphohyperplastic syndrome was diagnosed three months after detection of hematological abnormalities.

The mean follow-up duration was 2 ± 1 years. Among the survivors (85%), those with the involvement of the neuromuscular junction exhibited the most favorable prognosis. Regarding patients diagnosed with MG (*n* = 4), three demonstrated an improvement with initial therapy. All of these patients received oral prednisone for MG stabilization (Table 3). The three patients presenting with LEMS improved after initial therapy without subsequent neurological events. Conversely, patients presenting with LE experienced a less favorable prognosis, as only one of them exhibited signs of improvement. Patient #2 exhibited an early improvement in cognitive function in contrast to patient #10 who displayed an epileptic crisis and a behavioral disorder, necessitating adjustments of the antiepileptic therapy and oral therapy with prednisone (Table 3). Patient #3 remained stable during the follow-up period without further deterioration in cognitive dysfunction, while patient #13, who presented with cerebellar ataxia exhibited no improvement following treatment. Patient #6, who was diagnosed with overlap syndrome, demonstrated no improvement in cognitive function despite treatment (Table 3). The two individuals who died at 6 and 12 months after the initial diagnosis (15%), were patient #9 with anti-Hu and small-cell lung cancer and patient #7 with anti-Yo and gastric adenocarcinoma, respectively (Table 3). Notably, patient #7 demonstrated neurological improvement prior to tumor complications, while patient #9 experienced a rapid worsening over a three-month period, ultimately succumbing to tumor progression (Table 3).

## 4. Discussion

The present study provides a comprehensive overview of our single-center experience over an 8-year period, presenting thirteen representative PNS cases (eleven PNS-Abs positive and two PNS-Abs negative). These cases exhibit diverse clinical manifestations and distinct radiological presentations, reflecting the known clinical heterogeneity of PNS. With respect to clinical phenotypes, it is noteworthy that in line with relevant literature, there appears to be a positive correlation of PNS with advancing age, with a mean patient age of 63 ±14 years at symptom onset in the present cohort, without evidence of a sex predisposition [17]. Among the observed clinical syndromes, our findings demonstrate that neuromuscular junction disorders were the most frequent PNS, accounting for over half of the cases, followed by LE [18,19]. It should be noted, however, that there are disparities in the literature on whether MG may be considered one of the PNS, due to the established association of MG with benign underlying thymomas, with the revised 2021 PNS-CARE diagnostic criteria exempting MG from the PNS, in contrast to the European Federation Neurological Society guidelines [5,14]. Notably, the synchronous diagnosis of an underlying tumor was most frequent in neuromuscular junction disorders, while in the remaining cases, tumors were diagnosed prior to and within 6 months of PNS manifestation in 1 (8%) and 5 patients (38%), respectively.

Thymoma and small-cell lung carcinoma emerged as the most prevalent underlying tumors in our cohort, consistent with previous reports [20,21]. With respect to the identified PNS-Abs, anti-AChR (*n* = 4), anti-P/Q -VGCC (*n* = 3), anti-Hu (*n* = 3), anti-Yo (*n* = 1), anti-Ma2 (*n* = 1), anti-striational (*n* = 1), anti-IgLON5 (*n* = 1), and anti-GAD65 (*n* = 1) were detected with decreasing frequency. In line with existing literature, the simultaneous appearance of anti-striational (anti-Ti) and AchR antibodies in our study raises intriguing associations, particularly with underlying thymoma [22]. In addition, taking the prognostic implications of PNS-Abs into account, it is noteworthy that the survival rate in our cohort was exceptionally high, with only two patients deceased at a mean follow-up period of 2 years. These two patients exhibited Hu and Yo antibodies, that have been previously associated with poor survival and higher mortality rates in the literature [17].

From an epidemiological perspective, evidence from population-based cohorts indicates that PNS manifest at rates ranging from 0.25% to 3% among cancer patients seeking care at tertiary care referral centers, with a population-based incidence ranging from 2 to 3 cases per million person-years [21]. There is thus a strong disparity among cases that will be referred and consequently diagnosed at large tertiary or academic referral centers in comparison to community hospitals. With respect to PNS prognoses, the high survival rate recorded in our cohort may be attributed to the prompt suspicion of PNS and early treatment initiation, but also to the accessibility of PNS-Abs testing at tertiary academic hospitals, which may facilitate an earlier diagnosis. Apart from the type of implicated PNS-Abs, a prognosis is closely related to the underlying tumor, as indicated by the fulminant tumor progression experienced by patient #9 diagnosed with small-cell lung carcinoma [23]. Furthermore, the poor outcome of patient #7, diagnosed with gastric adenocarcinoma, highlights the impact of tumor-related complications common in gastric cancer, including impaired feeding and absorption [24]. It is noteworthy that despite the type of underlying malignancy, delays in tumor detection are linked to poor outcomes; thus, repetitive tumor screening in PNS is advisable to detect occult underlying malignancies [25]. Concerning applied treatments, there is still debate in the literature on whether immunosuppression for PNS may influence tumor outcomes [26]. Optimal immunotherapy remains debated, with immunomodulatory therapies, such as glucocorticoids, IVIG, and PLEX, as indicated in this cohort, often prioritized over B-cell or T-cell depleting therapies, due to fear of tumor spread and other related complications [26].

The present study also demonstrates that PNS with central nervous system involvement may appear with distinct radiological findings. Notably, abnormalities in brain imaging were observed only in two patients (15%) in accordance with the relevant literature that suggests that only a minority of PNS patients exhibit abnormalities in brain imaging [27]. In one patient, who presented with LE, typical imaging findings were reported in limbic areas, while the resolution of imaging abnormalities correlated with the remission of clinical symptoms following treatment. By contrast, one patient who presented with cerebellar ataxia and cerebellar atrophy during initial brain imaging, showed no evidence of clinical or radiological improvement following treatment. This case underscores that the reversal of clinical and radiological findings is highly dependent on the timing of treatment initiation (i.e., once cerebral atrophy is established, deficits may likely be irreversible) [28].

Concerning the diagnosis of PNS, there has been a recent shift in the diagnostic criteria from the 2004 to the recently revised 2021 PNS-Care criteria [3,5]. The present cohort allowed us to assess the comparability of these criteria with their implications on clinical practice. In particular, patient #3 and patient #10 of the present cohort presenting with IgLON5 and GAD65 antibodies, respectively, fulfilled the 2004 diagnostic criteria for a definite PNS diagnosis (Table 1) [3,29,30]. By contrast, due to the fact that IgLON5 and GAD65 are currently not considered PNS associated antibodies, these two cases were classified as non-PNS according to the 2021 PNS-Care criteria (Table 1) [5]. In addition, three additional patients who fulfilled the 2004 diagnostic criteria for definite PNS, were classified as probable PNS based on the revised 2021 PNS-Care criteria (with scores of 6 and 7, respectively; Table 1) [3,5]. The observed discordances in case classification indicate the higher specificity of the 2021 PNS-Care criteria, that mandate a causal association of detected antibodies with an underlying tumor for a definite PNS diagnosis, either based on evidence from relevant literature or histological studies [5]. To this end, it should be noted that the 2021 PNS-Care criteria were devised to facilitate the differentiation of PNS from immune-related adverse events (irAEs), which may manifest in association with oncological immunotherapies [31,32,33]. In this context, neurological syndromes arising in oncological patients undergoing immunotherapies (e.g., with immune checkpoint inhibitors) should be promptly differentiated, as different therapeutic approaches are required for PNS or irAEs treatment; thus, a high specificity of diagnostic criteria is essential.

The findings of our cohort align with the recent literature, including the study by Cai et al., 2022, that provides insight into the clinical application of the two different sets of diagnostic criteria [34]. Accordingly, it should be highlighted that although the 2021 PNS-Care criteria are characterized by higher specificity compared to the 2004 criteria, they are also characterized by a lower sensitivity [3,5]. In particular, the presence of a typical clinical phenotype, well-characterized PNS-Abs and/or an underlying tumor are prerequisites for a PNS diagnosis; a fact that vice versa precludes PNS diagnosis in patients with low-risk Abs or Abs not causally associated with an underlying tumor [5]. Nonetheless, with growing evidence from PNS research, our understanding of potential associations between Abs and underlying tumors along with their differentiation in high- vs. low-risk Abs continues to grow. Therefore, the lower diagnostic sensitivity of the revised 2021 PNS-Care criteria based on currently available evidence may also have detrimental effects, increasing the risk of under- or misdiagnosis (i.e., misclassification as probable/possible PNS) or causing significant delays in treatment initiation [5]. In view of the prognostic implications and in line with the previous literature, the long-term follow-up and monitoring of patients with probable/possible PNS is warranted to establish causal associations between detected Abs and underlying tumors [5,34]. We recommend weighing the harms and benefits of early vs. delayed treatment initiation in cases of probable/possible PNS on a per patient basis.

Certain methodological shortcomings of the present report need to be acknowledged. First, methodological limitations associated with the retrospective study design and the small sample size of our cohort need to be taken into account when interpreting our results. Thus, validation of the present results in larger prospective studies is warranted. Second, the diagnostic work-up was decided on a per patient basis; thus, standardized diagnostic algorithms (e.g., encompassing repeated CT or PET imaging and PNS-Abs testing) for tumor screening and follow-ups should be assessed in prospective studies. Third, previous studies have suggested that PNS may precede tumor manifestation by several years; thus, longitudinal studies with longer follow-up periods are required to establish the association between detected PNS-Abs and underlying tumors.

In conclusion, our study highlights that (i) the clinical presentation of PNS may be multifaceted; (ii) a prompt and thorough diagnostic work-up should be instigated for underlying malignancy detection; and (iii) the early suspicion of PNS decisively influences patient prognosis, with diagnosis and treatment delays accounting for the excess risk of PNS mortality as opposed to the overall good prognosis recorded in our patient collective. Future research should focus on refining the diagnostic and treatment algorithms of PNS with the aim to expand our understanding of these rare neurological syndromes and improve the clinical prognosis of PNS patients.

## Figures and Tables

**Table 1:** Recommended diagnostic criteria for paraneoplastic neurological syndromes (J Neurol Neurosurg Psychiatry 2004) in correspondence with the updated PNS-CARE diagnostic criteria for paraneoplastic neurological syndromes (Neurol Neuroimmunol Neuroinflamm 2021) [3,5].

2021 PNS-Care Criteria		2004 Graus-Criteria	
Criteria	Score	Criteria	
**Clinical Phenotype Risk Level** High-risk phenotype (syndrome often triggered by cancer) Intermediate-risk phenotype (can occur with or without cancer) Low-risk phenotype (weaker association with cancer) **Laboratory level** High-risk antibody (>70% cancer association) Intermediate-risk antibody (30–70% cancer association) Low-risk antibody (<30% cancer association) **Tumor** Identified, consistent with phenotype and antibody Not identified or not consistent with phenotype, with follow-up <2 years Not found, and follow up >2 years	3 2 1 3 2 0 4 1 0	Classical Syndrome	Non-classical syndrome
		Tumor present or tumor absent	
		PNS Abs present or absent	Improvement after therapy or PNS Abs present
		or well characterized PNS Abs present	
**Score**	**Diagnosis**	**Diagnosis**	
≥8	Definite PNS	Definite	
6–7	Probable PNS		
4–5	Possible PNS		
<4	Not PNS		

*Definite 2004 Graus criteria:* (1) classical syndromes characterized by the development of cancer, within 5 years of their initial diagnosis; or (2) non-classical syndromes diagnosed within 5 years of a cancer diagnosis, with the presence of PNS-Abs; or (3) non-classical syndromes showing substantial neurological improvement following cancer treatment; or (4) either classical or non-classical syndromes in conjunction with the identification of well-characterized PNS-Abs. *2021 PNS-Care criteria:* The scoring system integrates clinical phenotypes, antibodies, and tumor identification. Phenotypes are stratified as high or intermediate risk, based on distinct features. The scoring system classifies PNS-Abs as high, medium, or low risk. Cancer presence, aligned with the identified antibody within 2 years, is considered. A definitive PNS diagnosis (score ≥ 8) requires a high- or intermediate-risk phenotype, a corresponding antibody, and cancer presence. Abbreviations: PNS: paraneoplastic neurological syndromes; Abs: antibodies.

**Table 2:** Clinical characteristic, laboratory, and neuroimaging findings of patients presenting with paraneoplastic neurological syndromes. Evaluation by use of the 2004 Paraneoplastic Neurological Syndrome Classification and the revised 2021 PNS-Care Diagnostic criteria [3,5].

Patient	Sex	Age	Syndrome	Antibody	Tumor	CSF Analysis	MRI Brain	2004 Score	PNS Score
1	F	50	Myasthenia gravis	AchR, Ti	Thymoma	N/A	Normal	N/A	N/A
2	F	56	Limbic encephalitis	Ma2	Papillary thyroid carcinoma	normal	Normal	Definite	7
3	M	87	Limbic encephalitis	ΙgLON5	Adenocarcinoma of prostate	normal	Small vessel disease	Definite	3
4	F	64	LEMS	P/Q-VGCC	Small-cell lung carcinoma	N/A	Normal	Definite	9
5	F	70	Myasthenia gravis	AchR	Thymoma	N/A	Normal	N/A	N/A
6	M	80	LEMS, Limbic encephalitis	Hu, P/Q-VGCC	Small cell lung carcinoma	Increased total protein level	T2/FLAIR hyperintensity in left mesial temporal lobe	Definite	10
7	F	85	Cerebellar Ataxia	Yo	Gastric adenocarcinoma	Increased total nucleated cell count & elevated total protein level	Greater degree of iron deposition in the posterior-lateral part of the lenticular nuclei and cerebellar atrophy	Definite	6
8	M	77	LEMS	Hu, P/Q-VGCC	Non- small cell lung carcinoma	N/A	Normal	Definite	10
9	M	78	Peripheral neuropathy	Hu	Small-cell lung carcinoma	Elevated total protein level	Normal	Definite	10
10	M	27	Limbic encephalitis	GAD65	Lymphohyperpla-stic disorder	Increased total nucleated cell count	Normal	Definite	3
11	F	60	Myasthenia gravis	AchR	Thymoma	N/A	Normal	N/A	N/A
12	F	50	Myasthenia gravis	AchR	Thymoma	N/A	Normal	N/A	N/A
13	M	50	Cerebellar Ataxia	Yo	Papillary thyroid carcinoma	Normal	Cerebellar atrophy	Definite	6

Abbreviations: M = male; F = female; AchR = muscle-type acetylcholine receptor binding antibodies; Ti = titin; GAD = glutamic acid decarboxylase; P/Q-VGCC = P/Q type voltage-gated calcium channel (VGCC) antibodies, LEMS = Lambert–Eaton myasthenic syndrome; N/A = not applicable.

**Table 3:** Clinical characteristics of patients presenting with paraneoplastic neurological syndromes and responses to treatment, clinical course, and mean-elapsed time between neurological manifestation and the diagnosis of a tumor.

Patient	Syndrome	Antibody	Therapy	Response to Therapy	Course	Time to Tumor Diagnosis
1	Myasthenia gravis	AchR, Ti	Prednisone p.o., Pyridostigmine, Surgery	Improvement	Monophasic	3 months
2	Limbic encephalitis	Ma2	Antiepileptic drug (lacosamide), Surgery	Improvement in narcolepsy, minor problems in memory	Monophasic	2 years
3	Limbic encephalitis	ΙgLON5	Prednisolone i.v.	Stable	Monophasic	1 year
4	LEMS	P/Q-VGCC	IVIg, Pyridostigmine, Chemotherapy, Surgery	Improvement	Monophasic	1 year
5	Myasthenia gravis	AchR	Prednisone p.o., Surgery, radiotherapy	Improvement	Monophasic	1 month
6	LEMS, Limbic encephalitis	Hu, P/Q-VGCC	Prednisone p.o., Chemotherapy	Stable	Monophasic	1 week
7	Cerebellar Ataxia	Yo	IVIg, prednisolone i.v., surgery	Improvement	Death	1 month
8	LEMS	Hu, P/Q-VGCC	IVIg, amifampridine, surgery	Improvement	Monophasic	2 months
9	Peripheral neuropathy	Hu	IVIg, chemotherapy	Worsening	Death	2 weeks
10	Limbic encephalitis	GAD65	Prednisone p.o., antiepileptic drug (lacosamide)	Improvement in 1st year with gradual worsening in behavioral disorder and epileptic seizures	Relapsing	20 days
11	Myasthenia gravis	AchR	Plasmapheresis, IVIg, Prednisone p.o., cyclophosphamide, mycophenolate mofetil, surgery	Improvement	Relapsing	3 months
12	Myasthenia gravis	AchR	IVIg, Prednisone p.o., Surgery	Improvement	Monophasic	1 month
13	Cerebellar Ataxia	Yo	Prednisolone i.v.	Stable	Monophasic	1 year

Abbreviations: AchR = muscle-type acetylcholine receptor binding antibodies; Ti = titin; GAD = glutamic acid decarboxylase; P/Q-VGCC = P/Q type voltage-gated calcium channel (VGCC) antibodies; LEMS = Lambert–Eaton myasthenic syndrome; p.o. = per os; i.v. = intravenous; IVIg = intravenous immunoglobulins.

## Data Availability

The data presented in the manuscript are available from the corresponding author upon reasonable request.
